# The CalFresh Fruit and Vegetable EBT Pilot Project: A Model for Direct Nutrition Incentive Integration for SNAP Participants in the Retail Grocery Store Setting

**DOI:** 10.1177/15248399251394662

**Published:** 2025-12-10

**Authors:** Joe Prickitt, Adriana Bearse, Karemi Alvarez, Trixy Joy Manansala, Nancy Knauer, Eli Zigas, Grecia Marquez-Nieblas, Kyung E. Rhee, Sarah Hiller-Venegas, Blanca Melendrez

**Affiliations:** 1 Center for Community Health, Altman Clinical and Translational Research Institute, University of California San Diego, La Jolla, CA, USA; 2 Mother’s Nutritional Center, Santa Fe Springs, CA, USA; 3 Fullwell, Oakland, CA, USA; 4 University of California San Diego, La Jolla, CA, USA

**Keywords:** nutrition incentives, SNAP, CalFresh, food security, fruit and vegetable access, Electronic Benefit Transfer (EBT), retail grocery stores, public health nutrition, community partnerships

## Abstract

According to the U.S. Department of Agriculture (USDA) Economic Research Service, 13.5% of U.S. households experienced food insecurity in 2023, representing a notable increase from the previous year and emphasizing the urgent need for innovative enhancements to food assistance programs such as the Supplemental Nutrition Assistance Program (SNAP). This paper presents a case study of the CalFresh Fruit and Vegetable EBT (Electronic Benefit Transfer) Pilot Project, a pioneering initiative in California that enables SNAP recipients to earn a dollar-for-dollar rebate, up to $60 per month, on fresh fruits and vegetables purchased at participating grocery stores and farmers markets. Incentives earned through this program were automatically credited to recipients’ EBT accounts and could be redeemed for any SNAP-eligible foods at authorized USDA Food and Nutrition Service (FNS) retailers. At its launch, this was the only program in the U.S. to integrate nutrition incentives directly into SNAP EBT accounts for use at grocery stores. The Pilot Project achieved high satisfaction among both retailers and participants, resulting in nearly $18 million in incentives earned by approximately 93,000 SNAP recipients. This case study highlights key learnings and implications for practice, policy, and research and explores opportunities to replicate this model in other states and communities. Integrating nutrition incentives into the SNAP EBT system represents a promising strategy to promote healthier food choices and reduce food insecurity nationwide.

## Background and Program Overview

Lack of food security, defined as “access by all people at all times to enough food for an active, healthy life” (Economic Research Service, United States Department of Agriculture [USDA], 2025) remains a significant issue, affecting 18 million households in 2023 (Rabbitt et al., 2024). Food insecurity has been linked to higher risk of poor health outcomes (Sun et al., 2020). According to the Center on Budget and Policy Priorities, rising food insecurity across the United States demonstrates the need for policies that allow all families to afford nutritious meals (Nchako, 2024), particularly among children, single-parent households, and Black and Hispanic populations (Rabbitt et al., 2024).

The federal and state Supplemental Nutrition Assistance Program (SNAP) provides essential food assistance for millions of individuals and families with lower incomes each year (Center on Budget and Policy Priorities, 2024). However, SNAP does not specifically incentivize purchasing healthy foods like fruits and vegetables (Engel & Ruder, 2020). Many SNAP recipients face barriers to accessing fruits and vegetables due to cost (Engel & Ruder, 2020; Gearing et al., 2021).

Nutrition incentive programs have the potential to address this gap by making healthy foods more affordable, encouraging healthier choices, and helping participants stretch their food dollars. The USDA Healthy Incentives Pilot (HIP) implemented in Massachusetts in 2011–2012 demonstrated that nutrition incentives, such as rebates to SNAP recipients’ Electronic Benefit Transfer (EBT) accounts, could be successfully integrated into the EBT system to improve dietary choices and health outcomes for low-income individuals (Bartlett et al., 2014). Despite initial success, this program was only maintained at farmers markets in Massachusetts and, until 2023, had yet to be replicated or tested in retail settings elsewhere. Other nutrition incentive programs have been concurrently active across the United States; however, these programs have not been integrated directly into EBT systems (Byker Shanks et al., 2025).

This research brief describes key learnings and implications for practice, research, and policy from the CalFresh Fruit and Vegetable EBT Pilot Project (Pilot Project), a state-led initiative that directly integrated nutrition incentives into the SNAP EBT system in retail grocery stores across California. This paper responds to a critical need considering ongoing federal funding cuts to SNAP and EBT-related programs, emphasizing the urgency of identifying scalable solutions for nutrition access.

Building upon USDA HIP, the Pilot Project was launched to test the feasibility and effectiveness of delivering nutrition incentives directly through the SNAP EBT system in California, known as CalFresh. The Pilot Project was funded through an initial allocation of $9 million from the California general fund in 2018. Subsequent augmentations of $9.65 million (2023) and $10 million (2024) were also provided through the California general fund.

Historically, nutrition incentive programs at grocery stores have required separate vouchers, coupons, or loyalty cards, creating barriers to participation (Blumberg et al., 2022). To reduce barriers, the Pilot Project streamlined the earning and redemption of nutrition incentives by embedding a fully automated rebate mechanism into the CalFresh EBT system. Participation required no separate enrollment; when CalFresh recipients used their benefits to buy fresh fruits and vegetables at participating grocery stores or farmers markets, they instantly earned a dollar-for-dollar match, up to $60 per month. Incentives were credited to their EBT balances, rolled over month-to-month, and could be redeemed at any SNAP-authorized retailer for any SNAP-eligible foods.

While the Pilot Project operated in both farmers markets and grocery stores, this paper focuses on the grocery store implementation co-led by the University of California San Diego, Center for Community Health (UC San Diego), and SPUR/Fullwell.

### Innovative Approaches and Technologies

An essential feature of the Pilot Project was the integration of advanced technologies that facilitated real-time tracking of incentives and simplified the earning and redemption process for both participants and grocery retailers. To facilitate implementation, the California Department of Social Services (CDSS), in collaboration with the Office of Technology and Solutions Integration and its EBT vendor, Fidelity Information Services (FIS), conducted a series of technical upgrades to the state’s electronic benefit infrastructure. These upgrades enabled automated calculation and tracking of fruit and vegetable incentives for over 3 million CalFresh households (Alvarez et al., 2024).

Simultaneously, UC San Diego, SPUR/Fullwell, and participating grocery retailers including 79 Mother’s Nutritional Center stores across Southern California and 9 Northern California stores (Arteaga’s Food Center, 4 stores; Santa Fe Foods, 3 stores; and Harvest Market, 2 stores) worked with point-of-sale (POS) technology vendors to modify store systems. These modifications enabled transaction-level data to interface directly with California’s enhanced EBT system, supporting real-time nutrition incentive integration.

### Community Engagement and Partnerships

Findings from the USDA Healthy Incentives Pilot emphasized that strong community awareness and stakeholder engagement were critical components of incentive program success (Bartlett et al., 2014). Building on this, UC San Diego and SPUR/Fullwell partnered with nearly 500 community-based organizations (CBOs) statewide to increase visibility and participation. Outreach strategies included multilingual educational materials, coordinated social media, and direct engagement through local networks. County health departments, social service agencies, community centers, faith-based groups, and other safety-net organizations supported outreach efforts.

### Study Aims

The primary aim of this study was to assess the feasibility and effectiveness of delivering nutrition incentives directly through the SNAP EBT system in California. The UC San Diego Institutional Review Board determined this to be non–human subjects research (Protocol #804647).

1. Test the feasibility of integrating an automated nutrition incentive mechanism into the CalFresh EBT system.
2. Evaluate the model’s effectiveness in increasing fruit and vegetable purchases, achieving high participant satisfaction, and supporting implementation at retail grocery stores.
3. Document lessons learned from implementation with state agencies, technology vendors, retailers, and community partners.

### Methods

A mixed-methods evaluation was conducted in partnership with CDSS, participating retailers, and technology vendors.

### Measures

- *Feasibility measures* included (a) successful EBT/POS integration by site, (b) retailer-reported ease of implementation and operational fit, and (c) participant-reported awareness, ease of use, and barriers.
- *Effectiveness measures* included (a) self-reported changes in fruit and vegetable purchases, (b) perceived changes in diet/health, and (c) incentive redemption rate.

### Data Sources

#### Participant Surveys

CDSS offered a voluntary survey to CalFresh recipients who purchased qualifying fruits and vegetables at participating sites. After checkout, Pilot Project participants were given the opportunity to scan their receipt and complete a brief survey about shopping experiences and program perceptions. Approximately 6,000 incentive recipients participated in the survey; this Brief reports findings from five questions relevant to program aims, with item-level responses ranging from 3,860 to 3,880.

#### Retail Partner Surveys

A voluntary, self-administered survey was distributed to retail partner leadership and staff by UC San Diego, in collaboration with the Center for Nutrition & Health Impact. The survey assessed retailer preparedness, staff feedback, workflow impact, and alignment with store values.

#### Administrative EBT Data

California EBT transaction records tracked households, incentives earned and redeemed, and utilization. These data were generated by CDSS and FIS, then securely aggregated and shared with Pilot Project partners and retailers as de-identified summary reports to support implementation and evaluation.

### Analysis

Survey and administrative data were summarized with descriptive statistics. Redemption rate was calculated as the percentage of incentives redeemed. Open-ended survey responses were thematically coded.

### Evaluation Findings and Outcomes

#### Program Reach

Across two phases (February 2023–April 2024; October 2024–January 2025), approximately 93,000 CalFresh households participated, earning nearly $18 million in incentives. For context, California serves about 3 million CalFresh households. Participation represented about 3% of CalFresh households, limited by the project’s scale with 88 grocery stores and 7 farmers markets engaged.

#### Incentive Redemption

Administrative data showed a redemption rate of approximately 98%, compared with 55% in a prior paper-coupon program managed by SPUR/Fullwell.

#### Participant Survey Results

Most respondents (81%) reported increased fruit and vegetable purchases and found the Pilot Project EBT easy to use (80%). Nearly all (92%) perceived health improvements, and 95% supported program expansion (California Department of Social Services, 2024; Figure 1).

**Figure 1:**
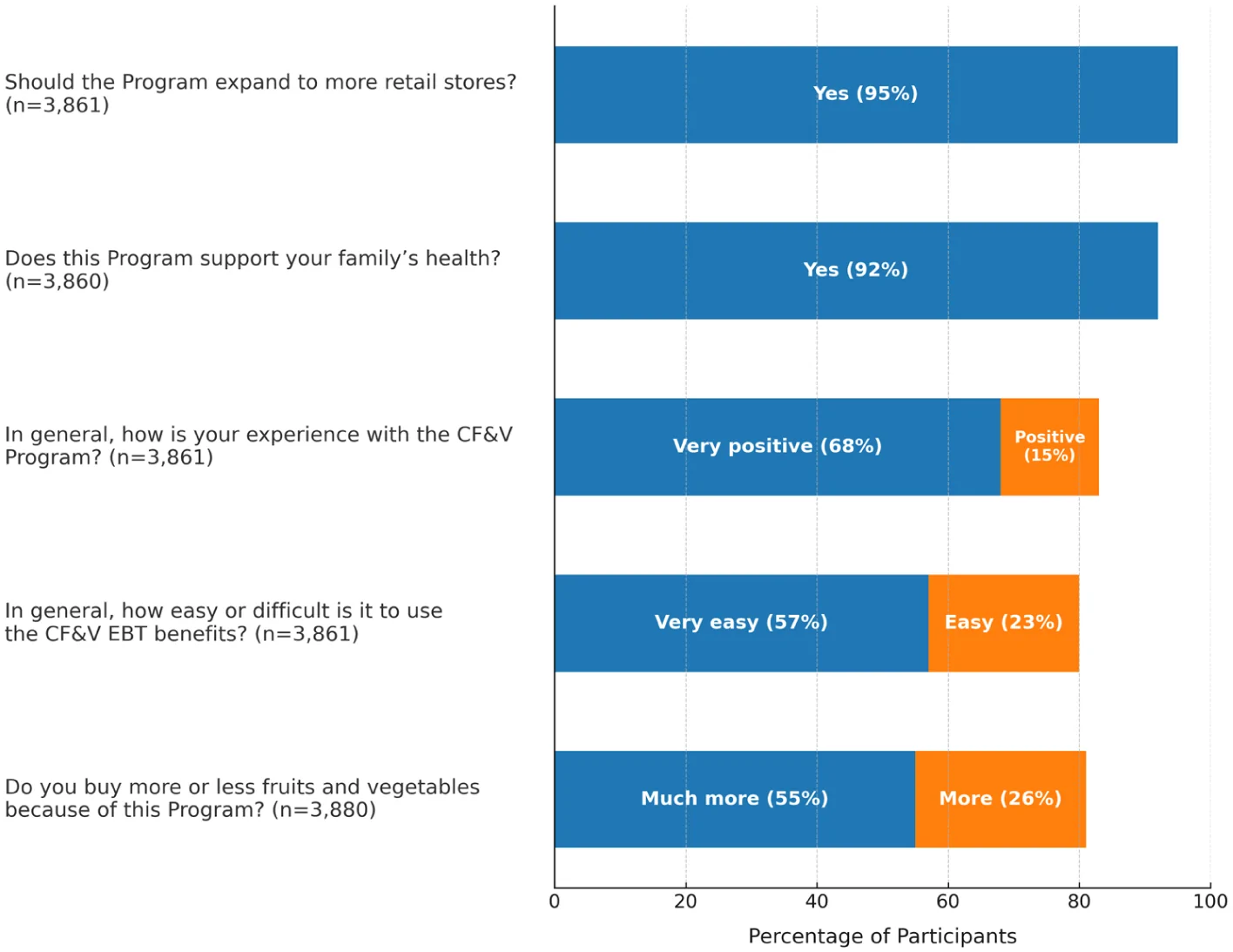
Participant Perceptions of the CalFresh Fruit and Vegetable EBT Pilot Program Source: Adapted from *Report for CF&V Customer Experience Survey* (Survey No. 6941225), by California Department of Social Services (2024). Unpublished manuscript.

#### Retailer Survey Results

Results indicated that the Pilot Project was easy to operationalize, aligned with store values, and beneficial to customer service; full analyses are reported separately (Houghtaling et al., forthcoming).

### Limitations

This study has several limitations. Participant survey data were collected through a voluntary, self-administered questionnaire that required scanning a QR code on transaction receipts. As such, findings may reflect self-selection bias and are not necessarily representative of all CalFresh participants who earned nutrition incentives. While retailer survey findings are summarized here, full methodological details and analyses are reported separately by an external evaluation team (Houghtaling et al., 2025). Structured interviews conducted by SPUR/Fullwell, with a handful of stores yielded results consistent with the UC San Diego evaluation. Due to its limited sample size, only the larger survey with Mother’s Nutritional Center retail staff and leadership (538 respondents; 89% response rate) is reported here. Together, these limitations underscore the importance of interpreting survey findings as exploratory and complementary to program transaction data, rather than as representative of the full population of participants or retailers.

### Key Learnings

Key learnings identified with Pilot Project partners, including retailers, county health departments, social service agencies, and CBOs, include the following:

1. **EBT integration increases utilization.** Participants were more likely to earn and redeem incentives when the process was simple at the point of sale, showing the value of EBT integration.
2. **Retailer partnerships are crucial.** Engaging retail partners in planning and implementation facilitated smooth integration. Collaboration on technology and training supported participation.
3. **Community-led outreach boosts uptake.** Partnerships with nearly 500 CBOs raised awareness among eligible households and expanded participation among groups facing access barriers.
4. **Long-term funding sustains impact.** Joint advocacy by retail and community partners secured $10 million in additional funding, allowing expansion across two cycles. Broader reach and impact will require ongoing, multi-year investment.

The Pilot Project’s structural approach of embedding nutrition incentives directly into the EBT system enabled participants to more easily earn and redeem benefits for SNAP-eligible foods. By reducing extra steps like separate enrollment or voucher redemption, this approach can address health equity barriers and enhance access to healthy foods for populations facing food insecurity.

The following implications for practice, research, and policy reflect lessons learned from the Pilot Project’s implementation experiences, partner feedback, and formative evaluation activities.

## Implications for Practice

The Pilot Project model can be readily adopted by USDA Food and Nutrition Service (FNS) authorized retailers of varying types and sizes. Key insights for replication include:

- Integrating incentives directly into EBT infrastructure, ensuring ease of use for participants and retailers.
- Building strong, sustained partnerships with retailers.
- Supporting retailers with training and system modifications needed to process EBT incentives.
- Working closely with community partners to raise awareness.

## Implications for Research

The Pilot Project provides a foundation for further evaluation and research, building upon existing literature on the effectiveness of nutrition incentive models (Bartlett et al., 2014). Future studies should assess:

- How financial incentives influence long-term purchasing behavior, diet quality including fruit and vegetable consumption, and health outcomes.
- Economic impact on retailers and local food systems.
- Scalability and sustainability of the technology infrastructure.

## Implications for Policy

The Pilot Project offers policymakers a scalable model to expand healthy food access for SNAP participants and strengthen local food economies. States can use this model to reduce food insecurity and diet-related disparities. To ensure long-term success, states should secure sustainable funding through budgets, federal grants, or private partnerships. Direct EBT integration reduces administrative and participation barriers, offering a structural approach that may improve equitable access to fruits and vegetables. Further evaluation should assess whether this approach narrows diet-related disparities.

## Conclusion

The CalFresh Fruit and Vegetable EBT Pilot Project modernizes nutrition assistance by integrating incentives directly into the SNAP EBT system. Its success stems from front-end simplicity, interoperability across multiple retailers, and stronger consumer and retailer participation compared to paper coupons or loyalty card models. California’s EBT/POS technologies are used in other states, upgrades are transferable. The model has been replicated in Washington, Rhode Island, Colorado, and Louisiana (Rhode Island Department of Human Services, 2024; National Institute of Food and Agriculture, U.S. Department of Agriculture, 2025). Policymakers should prioritize funding to expand and replicate this model as a permanent SNAP supplement.

## Data Availability

The data supporting the findings of this study are not publicly available due to participant confidentiality and agreements with partner organizations. Interested parties may contact the corresponding author to discuss potential access, subject to institutional review and approval.

## References

[bibr1-15248399251394662] Alvarez K. Prickitt J. Zigas E. Marquez-Nieblas G. Knauer N. Torres A. Bearse A. Melendrez B. (2024). CalFresh Fruit and Vegetable EBT Pilot Project: Perspectives from implementation in grocery stores. https://www.fullwell.us/articles/pilotreport 10.1177/15248399251394662 PMC13680729 41373178

[bibr2-15248399251394662] Bartlett S. Klerman J. Olsho L. Logan C. Blocklin M. Beauregard M. Enver A. & Abt Associates. (2014). Evaluation of the Healthy Incentives Pilot (HIP): Final report. U.S. Department of Agriculture, Food and Nutrition Service. https://www.fns.usda.gov/research/snap/hip/final-evaluation-report

[bibr3-15248399251394662] Blumberg R. Fowler E. Bai Y. Lal P. Smolen A. Dubrovsky I. (2022). An investigation of social ecological barriers to and facilitators of WIC Farmers Market Nutrition Program voucher redemption. Nutrients, 14(9), 1871. 10.3390/nu14091871 PMC9099787 35565837

[bibr4-15248399251394662] Byker Shanks C. Fung Uy W. Zhang N. Parks C. Fricke H. Resnicow K. Budd Nugent N. Yaroch A . (2025). Nutrition incentives associated with improved outcomes: 2020–2023 results from the U.S Gus Schumacher Nutrition Incentive Program. AJPM Focus, 4(4), 100348. https://www.ajpmfocus.org/article/S2773-0654(25)00036-7/fulltext 10.1016/j.focus.2025.100348 PMC12150091 40496549

[bibr5-15248399251394662] California Department of Social Services. (2024). Report for CF&V customer experience survey [Unpublished manuscript].

[bibr6-15248399251394662] Center on Budget and Policy Priorities. (2024). SNAP helps struggling families put food on the table. https://www.cbpp.org/research/food-assistance/snap-helps-struggling-families-put-food-on-the-table-0

[bibr7-15248399251394662] Economic Research Service, U.S. Department of Agriculture. (2025). Food security in the U.S. https://www.ers.usda.gov/topics/food-nutrition-assistance/food-security-in-the-us

[bibr8-15248399251394662] Engel K. Ruder E. H. (2020). Fruit and vegetable incentive programs for Supplemental Nutrition Assistance Program (SNAP) participants: A scoping review of program structure. Nutrients, 12(6), 1676. 10.3390/nu12061676 PMC7352438 32512758

[bibr9-15248399251394662] Gearing M. Dixit-Joshi S. May L. , & Westat Inc. (2021). Barriers that constrain the adequacy of Supplemental Nutrition Assistance Program (SNAP) allotments: Survey findings. Food and Nutrition Service, U.S. Department of Agriculture. https://fns-prod.azureedge.us/sites/default/files/resource-files/SNAP-Barriers-QualitativeReport.pdf

[bibr10-15248399251394662] HoughtalingB. MarriottJ. P. YarochA. L. PrickittJ. MelendrezB. AlvarezK. KnauerN. Byker ShanksC. (Forthcoming). Food retailers’ implementation of a fruit and vegetable nutrition incentive Electronic Benefit Transfer (EBT) Pilot Project in California. Translational Behavioral Medicine.10.1093/tbm/ibag02042114034

[bibr11-15248399251394662] National Institute of Food and Agriculture, U.S. Department of Agriculture. (2025). Gus Schumacher Nutrition Incentive Program (GusNIP) SNAP/NAP policy. https://www.nifa.usda.gov/gus-schumacher-nutrition-incentive-program-gusnip-snapnap-policy

[bibr12-15248399251394662] Nchako C. (2024). Food insecurity rises for the second year in a row. Center on Budget and Policy Priorities. https://www.cbpp.org/blog/food-insecurity-rises-for-the-second-year-in-a-row

[bibr13-15248399251394662] Rabbitt M. P. Reed-Jones M. Hales L. J. Burke M. P. (2024). Household food security in the United States in 2023 (Report No. ERR-337). Economic Research Service, U.S. Department of Agriculture. 10.32747/2024.8583175.ers

[bibr14-15248399251394662] Rhode Island Department of Human Services. (2024). SNAP eat well, be well pilot incentive program. https://dhs.ri.gov/programs-and-services/supplemental-nutrition-assistance-program-snap/supplemental-nutrition-8

[bibr15-15248399251394662] Sun Y. Liu B. Rong S. Du Y. Xu G. Snetselaar L. G. Wallace R. B. Bao W. (2020). Food insecurity is associated with cardiovascular and all‑cause mortality among adults in the United States. Journal of the American Heart Association, 9(19), Article e014629. 10.1161/JAHA.119.014629 PMC7792411 32975173

